# Characteristics of Particleboards Made from Esterified Rattan Skin Particles with Glycerol–Citric Acid: Physical, Mechanical, Chemical, and Durability Properties

**DOI:** 10.3390/polym18010107

**Published:** 2025-12-30

**Authors:** Mahdi Mubarok, Budi Arifin, Trisna Priadi, Yusuf Sudo Hadi, Deazy Rachmi Trisatya, Eko Setio Wibowo, Imam Busyra Abdillah, Resa Martha, Abdus Syukur, Obie Farobie, Lukmanul Hakim Zaini, Sukma Surya Kusumah, Philippe Gérardin, Holger Militz, Xiaojian Zhou, Ioanna A. Papadopoulou, Antonios N. Papadopoulos

**Affiliations:** 1 Department of Forest Products, Faculty of Forestry and Environment, IPB University, Bogor 16680, Indonesia; mahdimubarok@apps.ipb.ac.id (M.M.); trisnapr@apps.ipb.ac.id (T.P.); yshadi@indo.net.id (Y.S.H.); ibusyra.a@gmail.com (I.B.A.); abdssykr18@gmail.com (A.S.); lukmanhz@apps.ipb.ac.id (L.H.Z.); 2 LERMAB, INRAE, Université de Lorraine, 54000 Nancy, France; resa.martha@univ-lorraine.fr (R.M.); philippe.gerardin@univ-lorraine.fr (P.G.); 3 Department of Chemistry, Faculty of Mathematics and Natural Sciences, IPB University, Bogor 16680, Indonesia; budiarifin@apps.ipb.ac.id; 4 School of Forestry, Faculty of Natural Resources, Papua New Guinea University of Technology, Taraka Campus, Lae 411, Papua New Guinea; 5 Research Center for Biomass and Bioproducts, National Research and Innovation Agency (BRIN), Tangerang Selatan 15314, Indonesia; deaz001@brin.go.id (D.R.T.); ekos009@brin.go.id (E.S.W.); sukm002@brin.go.id (S.S.K.); 6 Department of Mechanical and Biosystem Engineering, Faculty of Agricultural Engineering and Technology, IPB University, Bogor 16002, Indonesia; obiefarobie@apps.ipb.ac.id; 7 Department of Wood Biology and Wood Products, Georg-August Universität Göttingen, 37073 Göttingen, Germany; hmilitz@gwdg.de; 8 College of Materials and Chemical Engineering, Southwest Forestry University, Kunming 650224, China; 9 Department of Chemistry, Aristotle University of Thessaloniki, 54124 Thessaloniki, Greece; papad.ioanna@chem.auth.gr; 10 Laboratory of Wood Chemistry and Technology, Department of Natural Environment and Climate Resilience, Democritus University of Thrace, 66100 Drama, Greece

**Keywords:** esterification, glycerol, citric acid, field test, instrumental analysis, particleboard, polyurethane, rattan skin

## Abstract

This study evaluated the effects of glycerol–citric acid (Gly-CA) modification and polyurethane (PU) adhesive concentration on the properties and termite resistance of rattan skin-based particleboards. Rattan skin particles were modified with 0%, 20%, and 40% Gly-CA and bonded using 6% or 12% PU adhesive. Gly-CA modification significantly improved dimensional stability, reducing water absorption and thickness swelling to about 35–40% and 4–6%, respectively, at 40% Gly-CA with 12% PU. However, excessive modification decreased mechanical strength due to over-crosslinking, while 20% Gly-CA provided the best balance between strength and stability. FTIR analysis confirmed ester and urethane bond formation, while thermogravimetric results showed enhanced thermal stability with increasing Gly-CA content (T_max_ up to 356.8 °C). Field tests conducted over 98 days revealed a substantial improvement in durability, with termite-induced mass loss decreasing from about 28% in untreated boards to below 8% in Gly-CA–modified samples. From this study, the combination of 40% Gly-CA modification and 12% PU adhesive produced particleboards with improved dimensional and thermal stability, as well as durability against termites. These findings highlight glycerol–citric acid bio-modification as a sustainable and effective strategy for developing durable eco-friendly rattan skin-based composites.

## 1. Introduction

Rattan is one of the natural resources widely utilized in the furniture and handicraft industries in Indonesia. As a country contributing 85% of global rattan production, Indonesia’s rattan output in 2023 reached 1.77 million canes, with 90% sourced from the islands of Kalimantan, Sumatra, and Sulawesi, and the remaining 10% from Java [1,2]. Despite this potential, approximately 28–40% of the rattan stem, particularly the outer skin, becomes underutilized waste during processing [3]. This rattan skin waste, however, contains high lignocellulosic content, making it suitable for value-added products such as particleboard. In line with Indonesia’s downstream development strategy to maximize the value of its natural resources, the utilization of rattan skin waste for particleboard production aligns with national industrial goals and circular economy principles [2,4].

As a composite product, one of the main challenges in using rattan skin as a raw material for particleboard is its unstable hygroscopic nature and vulnerability to degradation by biological agents [5]. Rattan skin has a high moisture content, making it susceptible to pests such as termites [5]. To enhance rattan skin’s resistance to moisture and biological deterioration, appropriate drying and chemical preservation treatments are required. The drying process aims to reduce the moisture content, minimizing fungal attacks, while chemical preservation can improve resistance to pest attacks, particularly insects. However, similar to wood, increasing awareness over the past two decades about the safety of preservatives and environmentally friendly preservation techniques, e.g., Schultz et al. [6], has led to greater interest in non-biocidal preservation technologies for lignocellulosic materials [7,8,9,10,11,12,13,14,15,16,17,18,19,20,21,22,23,24,25,26,27,28,29]. Although the properties of particleboard, especially rattan-skin-based boards, are influenced by the type and amount of adhesive used, increasing the adhesive content in industrial applications does not significantly alter the inherent characteristics of rattan skin particles. Therefore, a method is needed to improve the quality and durability of rattan skin particles so that the resulting particleboard has better performance.

As a lignocellulosic material, rattan skin particles share similar components with wood particles. Thus, methods to enhance their quality will also be similar to those used for wood. Traditional wood enhancement methods using biocide chemicals such as borax, creosote, penta, permethrin, CCA, and others are now being phased out and will soon be banned [30], necessitating eco-friendly alternatives. One such solution is lignocellulosic material modification, both for wood and rattan skin. Thermal and chemical modification techniques have been widely developed and even industrially implemented to improve the durability and dimensional stability [14]. Thermal modification has been studied for decades and marketed for its benefits in overcoming dimensional instability without the use of harmful chemicals [31,32]. However, it may cause cellulose depolymerization and loss of hemicellulose, leading to reduced mechanical strength [33]. Moreover, the resulting materials still have low termite resistance, especially in ground contact conditions [11,34]. On the other hand, chemical modification involving the impregnation of active substances into lignocellulosic materials offers an alternative to address the limitations of thermal modification. Although many studies on chemical modification have been conducted [14,20,35,36,37], including research on wood esterification [10,13,16,17,23,24,25,29] and polymer impregnation [12,13,15,18,22,29,30,38,39,40], only a few techniques such as acetylation, furfurylation, and DMDHEU (1,3-dimethylol-4,5-dihydroxyethyleneurea) processes have been applied industrially [14,35,41,42].

Among the various chemical modification approaches, environmentally friendly chemical-based lignocellulosic modification is seen as more promising. Polyesterification using glyceryl citrate is one such method that can be applied to improve the quality of lignocellulosic materials [13], including rattan skin in this case. This polyesterification process is expected to reduce the hygroscopicity of the material, enhance resistance to biological degradation, and improve dimensional stability, making it more suitable as a raw material for stable particleboard. The development of glyceryl citrate polyesterified rattan-skin-based particleboard is expected to serve as an innovative solution for reducing rattan industry waste while producing higher-performing products, particularly in terms of dimensional stability. Additionally, utilizing rattan skin waste supports circular economy principles and sustainability in Indonesia’s forestry and wood industries.

This study aims to synthesize particleboard from rattan skin previously modified with the environmentally friendly chemical glycerol–citric acid at varying levels of modification. The chemical, physical, and mechanical properties of the resulting particleboards will be analyzed. Furthermore, the termite resistance of the boards under field tests will also be evaluated to determine the overall performance of the modified rattan-skin-based particleboard.

## 2. Materials and Methods

### 2.1. Materials

The materials used in this study included rattan skin obtained from Sentra Rotan Desa Tegalwangi, Cirebon, West Java, Indonesia; glycerol and citric acid obtained from Sigma Aldrich; and polyurethane/PU adhesive, which was based on the mixture of methylene diphenyl diisocyanate/MDI (U7AA240978) and polyol blend (PB/95170625) obtained from PT Anugrah Raya Kencana, Serang, Indonesia. The equipment used included a dish particle machine, caliper, camera, scanner, digital balance, oven, universal testing machine (UTM), impregnation container, glue–particle mixer, hot-press machine, circular saw, Fourier transform infrared (FTIR) spectrometer, and a thermogravimetric analyzer (TGA).

### 2.2. Poly-Esterification of Rattan Skins

Rattan skins were first cleaned to remove foreign matter and then chopped and ground into flakes using a dish-flaker machine. The obtained flakes, measuring 2–5 cm in length and 0.2–0.5 cm in width, were then dried in an oven at 60 °C until they reached a moisture content of less than 10%. The dried rattan skin flakes were then immersed in an impregnating solution containing a 20% or 40% aqueous mixture of glycerol and citric acid with a molar ratio of 1:1 for 2 h. The impregnated rattan skin flakes were then drained to remove the excess impregnating solution and dried at 60 °C for 24 h and at 103 °C for 48 h. The dried rattan skin flakes were then polymerized in an oven at 150 °C for a 4 h heating process, loosely covered with aluminum foil. The Gly-CA content (20% or 40%) and curing condition were selected based on prior similar studies, where CA-based modification yielded optimal crosslinking within this concentration range and condition for lignocellulosic materials [10,13]. The polyesterified rattan skin flakes were then used to make particleboard.

### 2.3. Particleboard Production

Polyesterified rattan skin flakes were first mixed with polyurethane (PU) adhesive made from the mixture of methylenediphenyl diisocyanate (MDI) and polyol (1:1) (resin solid content = 95%). Based on the oven-dry weight of rattan skin particles, the solid adhesive concentration used was 6% or 12%, which falls within the typical range applied in industrial particleboard production. The adhesive–flakes mixture was then mat-formed and hot-pressed at 150 °C (an appropriate temperature for curing PU-based adhesives) for 10 min under 25 kg/cm^2^ specific pressure (a value commonly used in industrial particleboard manufacturing), so that the final dimension of the particleboard was 300 mm × 300 mm × 10 mm. The obtained particleboards were then conditioned at room conditions (at 27–30 °C and 60–70% of relative humidity) for two weeks before testing.

### 2.4. Characterization

#### 2.4.1. Physical Properties

##### Moisture Content

The moisture content of the particleboards was determined using the oven-dry method according to JIS A 5908:2003 [43]. Test specimens (*n* = 3) with dimensions of 50 mm × 50 mm × 9 mm were randomly taken from each board. Each sample was first weighed to obtain the initial weight (W_1_) and then oven-dried at 103 ± 2 °C until a constant weight was reached. After cooling in a desiccator, the oven-dried weight (W_0_) was recorded. The moisture content was calculated using the following equation:

(1) $$\mathrm{MC}\left(\%\right)\;=\;\frac{W_{1}-\;W_{0}}{W_{0}}\;\times \;100.$$

Measurements were conducted with six replications per treatment, and the results were expressed as the mean values. This procedure followed JIS A 5908:2003 [43], which has also been applied in previous studies by Karliati et al. [44], Sutiawan et al. [45], Syahfitri et al. [46], and Syukur et al. [47].

##### Density

The density of the particleboards was measured according to JIS A 5908:2003 [43]. Specimens (*n* = 3) with dimensions of 50 mm × 50 mm × 9 mm were cut from each board after a two-week conditioning period. The density (ρ) was calculated by dividing the oven-dried mass (M) of each specimen by its volume (V), using the following equation:

(2) $$\rho \;\left(\mathrm{kg}/m^{3}\right)\;=\;\frac{M}{V},$$

where M is the oven-dried weight (kg), and V is the specimen volume (m^3^), obtained from its measured length, width, and thickness. Each treatment was tested with six replications, and the average values were reported. The procedure followed JIS A 5908:2003 [43] and is consistent with previous studies on particleboards reported by Karliati et al. [44] and Sutiawan et al. [45].

##### Color Characteristics

The surface color of the particleboards was determined using the CIELab method by measuring L* (lightness), a* (red to green), and b* (blue to yellow) values, with a scanner (CanoScan 4400F, Canon, Tokyo, Japan) and Adobe Photoshop CS5. The color change (ΔE) of the specimens (*n* = 10) was also calculated, based on the CIELab method, while classification followed Hunter Lab [48] and Hadi et al. [49]. The color parameters were expressed in the CIE Lab system*, where L represents lightness (0 = black, 100 = white), a indicates the green–red axis, and b represents the blue–yellow axis. Measurements were performed on three different points on the surface of each specimen, and the average values were recorded. The total color difference (ΔE*) between the untreated and modified particleboards was calculated using the following formula:

(3) $$∆E^{*}=\sqrt{{\left(∆L^{*}\right)}^{2}+{\left(∆a^{*}\right)}^{2}+{\left(∆b^{*}\right)}^{2}}.$$

This method is widely used for assessing the color changes in wood and lignocellulosic composites after chemical modification or thermal treatment [50].

##### Thickness Swelling

The thickness swelling of the particleboards was measured according to JIS A 5908:2003 [43]. Specimens (*n* = 6) with dimensions of 50 mm × 50 mm × 9 mm were dried to a constant weight and thickness and then immersed in distilled water at 20 ± 2 °C for 2, 6, and 24 h. The specimen thickness was measured at the center of each panel using a digital caliper (±0.01 mm) before immersion (T_0_) and immediately after immersion (T_1_). The thickness swelling was calculated using the following equation:

(4) $$\mathrm{TS}\;\left(\%\right)\;=\;\frac{T_{1}-\;T_{0}}{T_{0}}\;\times 100.$$

Six specimens per treatment were tested, and the average values were reported. The procedure followed JIS A 5908:2003 [43], which has been widely adopted in previous studies on citric-acid-modified particleboards [44,50,51].

##### Water Absorption

The water absorption was determined simultaneously with the thickness swelling test according to JIS A 5908:2003 [43]. Specimens (*n* = 6) with dimensions of 50 mm × 50 mm × 9 mm were oven-dried to a constant weight (W_0_) prior to testing. The specimens were then immersed in distilled water at 20 ± 2 °C for 24 h. After immersion, excess water on the surface was removed using filter paper, and the wet weight (W_1_) was recorded. The water absorption was calculated using the following formula:

(5) $$\mathrm{WA}\left(\%\right)\;=\;\frac{W_{1}-W_{0}}{W_{0}}\;\times \;100$$

Six specimens per treatment were tested, and the mean values were reported. This method followed JIS A 5908:2003 [43], which has been commonly applied in previous studies on bio-based particleboards [44,50,51].

#### 2.4.2. Mechanical Properties

##### Modulus of Elasticity and Modulus of Rupture

The bending properties of the particleboards, namely the modulus of elasticity (MOE) and modulus of rupture (MOR), were evaluated following JIS A 5908:2003 [43], using a universal testing machine (UTM, Shimadzu AG-10 kN, Kyoto, Japan). The test specimens (*n* = 6) were prepared with dimensions of 200 mm × 50 mm × 9 mm (length × width × thickness) and conditioned for two weeks prior to testing. A three-point bending test was performed with a span length of 150 mm and a loading speed of 10 mm/min. The load–deflection data were recorded automatically, from which the MOE and MOR values were calculated according to the standard equations. Six replications were carried out for each treatment, and the average values were reported. This procedure is consistent with previous studies on particleboards conducted by Umemura et al. [51], Widyorini et al. [52], and Karliati et al. [44].

##### Internal Bonding

The internal bonding (IB) strength of the particleboards was determined in accordance with JIS A 5908:2003 [43], using a universal testing machine (UTM, Shimadzu AG-10 kN, Kyoto, Japan). Test specimens (*n* = 6) with dimensions of 50 mm × 50 mm × 9 mm were cut from the conditioned boards and bonded at the center to loading blocks with hot-melt adhesive. A tensile load was then applied perpendicular to the board surface at a crosshead speed of 2 mm/min until failure occurred. The maximum load at failure was recorded and divided by the specimen’s cross-sectional area to calculate the IB strength, expressed in N/mm^2^. One specimen per treatment was tested with six replications, and the mean values were reported. This procedure is consistent with the particleboard testing protocols used in previous studies [51].

##### Screw Holding Strength

The screw holding strength (SHP) of the particleboards was evaluated following JIS A 5908:2003 [43], using a universal testing machine (UTM, Shimadzu AG-10 kN, Kyoto, Japan). Test specimens (*n* = 6) with dimensions of 100 mm × 50 mm × 9 mm were prepared from conditioned boards. Standard wood screws (3.5 mm diameter × 30 mm length) were inserted vertically into the specimens to a depth of 15 mm at two positions per sample. The screws were then withdrawn at a crosshead speed of 2 mm/min, and the maximum withdrawal load was recorded. SHP values were expressed in Newtons (N). Six replications were carried out for each treatment, and the mean values were reported. This procedure followed JIS A 5908:2003 [43], which was consistent with previous studies on particleboard mechanical performance [44,50].

#### 2.4.3. Chemical Properties

##### Fourier Transform Infrared

Fourier transform infrared (FTIR) spectroscopy was conducted to characterize the chemical structure of untreated and Gly-CA-modified rattan flakes, as well as the resulting particleboards. Spectra were obtained using an FTIR spectrometer (Perkin Elmer Spectrum 2000, PerkinElmer, Inc., Überlingen, Germany) equipped with an attenuated total reflectance (ATR) accessory. Finely ground samples were scanned over the range of 4000–500 cm^−1^ at a resolution of 4 cm^−1^, with 16 scans per sample. The spectra were baseline corrected and normalized prior to analysis. The procedure followed standard practices commonly applied in studies of citric acid–glycerol-modified lignocellulosic materials [11].

##### Thermogravimetric Analysis

The thermal stability of the untreated and Gly-CA-modified rattan skin particleboards was analyzed using a thermogravimetric analyzer (TGA/DSC1-TMA/SDTA 84Xe instrument from Mettler Toledo equipped with the STARe v.11 fr System program, Nänikon, Switzerland). Approximately 10 ± 1 mg of finely ground samples were placed in a platinum pan and heated from 30 °C to 600 °C at a constant heating rate of 10 °C/min under a nitrogen atmosphere (50 mL/min) to avoid oxidative degradation. The onset temperature (T_0_), maximum degradation temperature (T_max_), and peak temperature obtained from the derivative thermogravimetric (DTG) curve and the residual mass at 600 °C were recorded for each sample. The results were compared to evaluate the influence of Gly-CA modification on the thermal behavior of the particleboards. This procedure followed established methods reported in previous studies on sorbitol–citric acid-modified bio-based composites [10].

#### 2.4.4. Field Test Study

**Figure 1:**
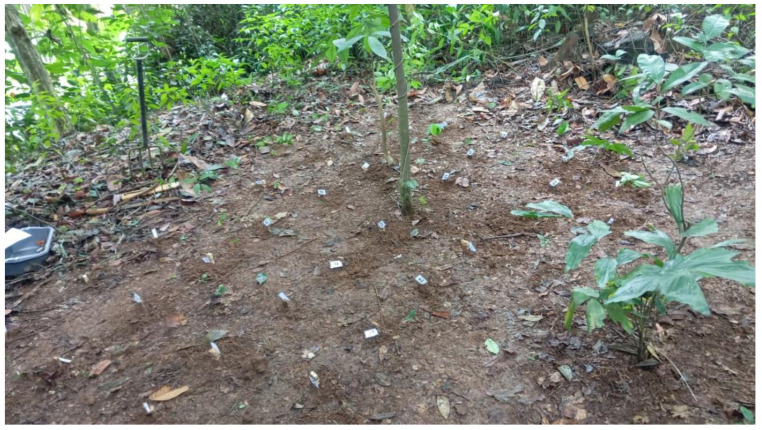
Field test site and appearance of particle boards placed on the ground.

The particleboards (*n* = 6) made from untreated and Gly-CA-modified rattan skin particles with different PU adhesive concentrations were cut into sticks measuring 200 mm × 20 mm × 9 mm, oven-dried at 103 °C for 48 h, and weighed to determine the initial weight before the field test. The samples were then buried in soil to a depth of 175 mm, leaving 25 mm above the ground surface. The in-soil exposure was considered a more rigorous durability test compared to above-ground exposure, with a total duration of 98 days (21 June–26 September 2025). All samples were randomly buried approximately 300 mm apart, with the untreated samples placed representatively at each corresponding location within the test field (Figure 1).

The field test was conducted at the Arboretum of the Faculty of Forestry and Environment, IPB University, Bogor, Indonesia. After 98 days, the samples were removed, washed with water, and brushed to remove soil or mud. They were then conditioned at room temperature and oven-dried again at 103 °C to a constant weight. The mass loss due to termite attack was calculated using the following formula:

ML (%) = 100 × (M_1_ − M_2_)/M_1_, (6)

where ML is the mass loss due to termite attacks, M_1_ is the initial dry mass of the sample before the field test, and M2 is the dry mass of the sample after the field exposure. The attack was evaluated according to E7-07 AWPA standard [53] (Table 1).

### 2.5. Data Analysis

To determine the effect of the treatments upon all the response variables, a completely randomized block design was used for data analysis. The block was the adhesive content, namely 6% and 12%, and the treatments were untreated, Gly-CA20%, and Gly-CA40%. Tukey analysis tests were used for further analysis if a factor was significantly different at *p* ≤ 0.05. The data were analyzed with Microsoft Excel and IBM SPSS Statistics version 27.

## 3. Results and Discussion

### 3.1. Physical Properties

The physical characteristics evaluated in this study included the density, moisture content, thickness swelling, and water absorption. Density, defined as the mass of material per unit volume, is often associated with the strength of a material. In general, higher-density materials exhibit superior physical and mechanical properties compared to those with lower density; however, this relationship is more evident in solid homogeneous materials. In non-homogeneous materials, density alone may not be a reliable indicator of mechanical performance unless accompanied by strong internal bonding within the structure.

**Figure 2:**
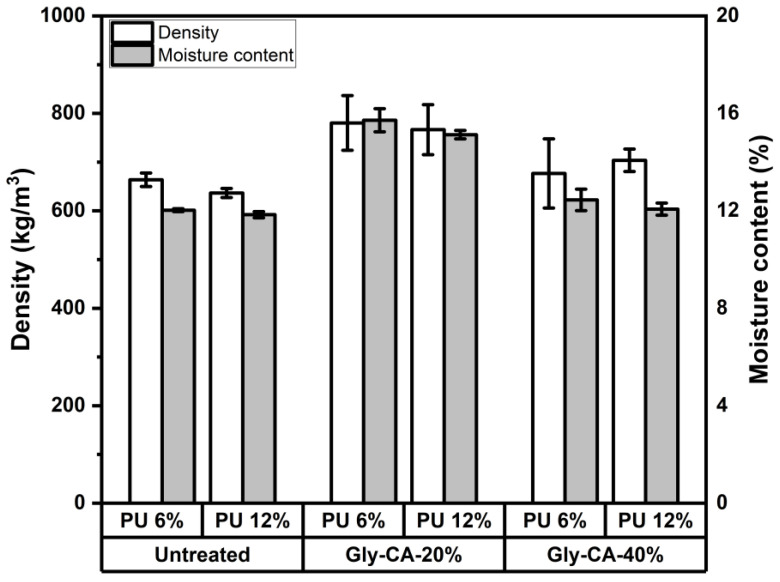
The density and the moisture content of particleboards made from different modification levels and adhesive concentrations.

In the present study, the density of the particleboard increased with higher Gly-CA concentrations (Figure 2). The ANOVA results indicated that modification significantly affected the density (*p* = 0.001), whereas the adhesive concentration (*p* = 0.823) and the interaction between the modification and adhesive concentration (*p* = 0.557) were not significant. The observed increase suggests that modification enhanced the density of the raw rattan flakes. As the quantity of the raw material used during particleboard production was constant across treatments, similar densities would be expected for all boards. Nevertheless, the significant effect of modification implies that the application of Gly-CA at 20% likely reduced the compression ratio of the rattan flakes, producing boards with higher density. This finding highlights the role of chemical modification not only in altering the surface chemistry but also in influencing the bulk physical characteristics of composite products.

Regarding the moisture content, the relevant standard specifies a maximum value of 14% to minimize the risk of decay, which becomes more likely when the moisture content exceeds 20%. In addition to biodegradation concerns, an elevated moisture content can negatively affect the physical properties of boards (e.g., dimensional instability) as well as the mechanical performance (e.g., reduced hardness, MOE/MOR, and bonding strength) [34]. In the present study (Figure 2), the moisture content of the particleboards ranged from 11.8% to 15.7%. The ANOVA results indicated that both the adhesive concentration (*p* = 0.018) and modification level (*p* < 0.001) had significant effects on the moisture content, whereas their interaction was not significant (*p* = 0.517). Among all specimens, the boards manufactured from Gly-CA 20%-modified rattan skin exhibited the highest moisture content (15.7%). Although this value slightly exceeded the standard limit, the increase is likely attributable to the higher initial moisture content of the Gly-CA 20%-modified rattan skin used during board production. Since the density of water is relatively higher than the bulk density of commonly lignocellulosic materials (e.g., wood, natural fibers, rattan), this higher moisture content might also contribute to the higher density of the boards.

**Figure 3:**
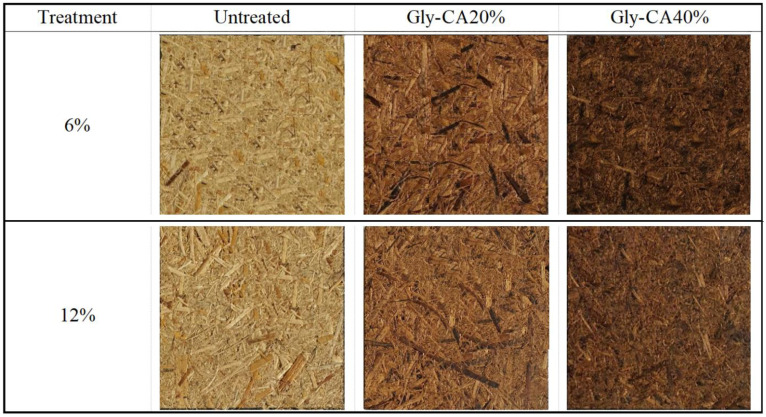
Particleboard, untreated and modified with Gly-CA.

The color characteristics of the particleboard treated with various resin contents and modified using glycerol–citric acid (Gly-CA) pretreatment are summarized in Table 2 and shown in Figure 3. The untreated particleboard showed the highest lightness (L*) value of 65.20, indicating a relatively bright appearance. However, with the Gly-CA pretreatment, a significant decrease in lightness was observed, with the Gly-CA-20% treatment at 6% resin content showing a value of 41.80 and the Gly-CA-40% treatment at 6% resin content further dropping to 23.00. This suggests that increasing the Gly-CA treatment results in a darker particleboard. The most plausible explanation is that higher Gly-CA content enhances esterification and thermal reactions, which promote caramelization and the formation of chromophoric compounds. A greater degree of crosslinking also leads to the increased thermal degradation of hemicelluloses and extractives, contributing to the observed color changes [10,11]. Consequently, these chemical modifications result in lower L* values (darkening) and also noticeable shifts in the a* and b* coordinates [40]. The redness–greenness (a*) values decreased dramatically with the Gly-CA treatments. The untreated sample exhibited an a* value of 6.60, while the Gly-CA-20% treatment reduced it to 1.30 at 6% resin and 3.78 at 12% resin, indicating a shift towards greener tones. The Gly-CA-40% treatment further lowered the a* value to 1.48 at 6% resin and 2.07 at 12%, showing a more pronounced shift toward green. For the yellowness–blueness (b*) values, the untreated particleboard had a high b* value of 33.40, indicating a yellowish tone. After Gly-CA treatment, the b* values decreased significantly, with the Gly-CA-20% treatment at 6% resin showing a value of 17.20, and the Gly-CA-40% treatment at 6% resin further reducing the b* value to 14.20. This indicates a shift away from yellow tones to more neutral colors. The overall color difference (ΔE) values, which indicate the extent of color change, were not observed for the untreated particleboard but were notably higher for the treated samples. The Gly-CA-20% treatment at 6% resin content showed a ΔE of 26.55, and the Gly-CA-40% treatments had even higher ΔE values, reaching 46.02 at 6% resin and 42.66 at 12% resin, highlighting the substantial impact of the Gly-CA pretreatment on the color characteristics of the particleboard. These findings indicate that the Gly-CA treatment and resin content significantly alter the color properties of the particleboard, making it visually distinct from the untreated samples. Further studies could explore the chemical mechanisms underlying these color changes and their potential implications for the material’s applications.

**Figure 4:**
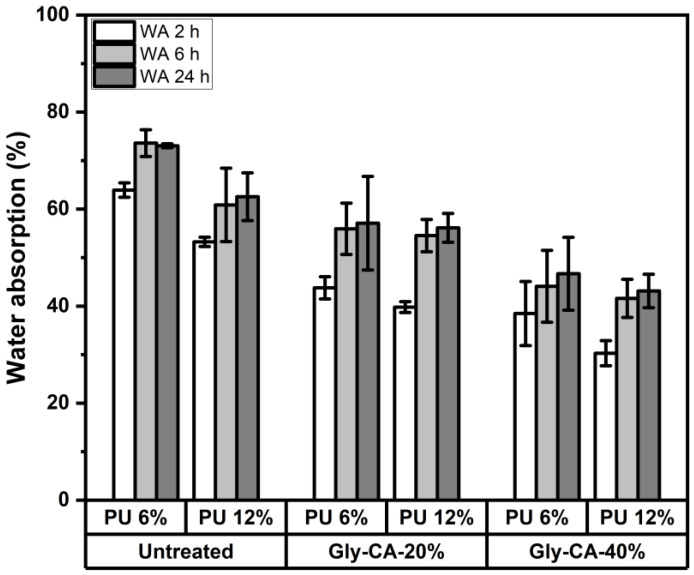
Water absorption characteristics of particleboards made from different modification levels and adhesive concentrations.

The liquid water absorption (WA) of the particleboards produced from untreated and glycerol–citric acid (Gly-CA)-modified rattan skin at different concentrations, bonded with polyurethane adhesive at two resin loadings, is presented in Figure 4. In all cases, the WA increased with prolonged immersion time (2, 6, and 24 h). However, clear differences were observed between the untreated and modified particleboards. Untreated rattan skin particleboards exhibited the highest WA values, reaching approximately 75–80% and 65% after 24 h for panels bonded with 6% and 12% adhesive, respectively. This high-water uptake is attributed to the hydrophilic nature of rattan skin fibers and the absence of chemical modification to restrict hydroxyl group activity. In contrast, Gly-CA modification significantly reduced the WA in comparison to the untreated samples. Particleboards modified with 20% Gly-CA displayed WA values in the range of 55–60% after 24 h, while 40% Gly-CA modification further reduced WA to about 40–50%, depending on the adhesive loading. This improvement is likely due to esterification reactions between the citric acid carboxyl groups and hydroxyl groups in rattan skin, which decrease the availability of hydrophilic sites and thereby hinder water penetration. The adhesive concentration also played a significant role in the WA behavior. Panels bonded with 12% adhesive consistently exhibited lower WA than those with 6% adhesive, regardless of the modification level. A higher adhesive content improves the bonding coverage and creates a denser polymer network, limiting voids and pathways for water diffusion. Overall, the lowest WA values (approximately 35–40% after 24 h) were achieved by particleboards produced from rattan skin modified with 40% Gly-CA and bonded with 12% adhesive. These findings indicate that combining chemical modification with sufficient adhesive loading effectively enhances the dimensional stability of rattan skin-based particleboards under wet conditions.

**Figure 5:**
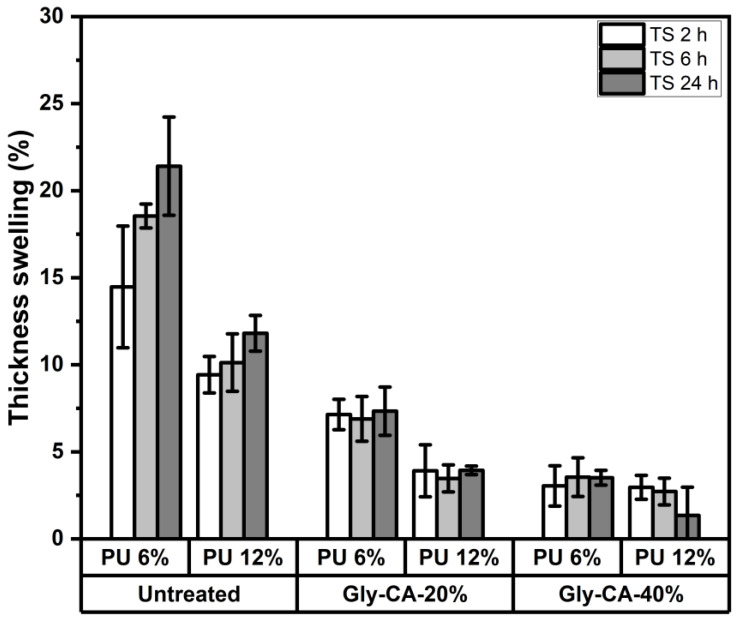
Thickness swelling characteristic of particleboards made from different modification levels and adhesive concentrations.

The thickness swelling (TS) of rattan skin particleboards after immersion for 2, 6, and 24 h is shown in Figure 5. Across all samples, the TS increased with the immersion time, but the magnitude varied significantly depending on whether the rattan skin was untreated or chemically modified, as well as on the adhesive loading. Untreated particleboards exhibited the highest TS values, with swelling reaching approximately 20–22% after 24 h for panels bonded with 6% adhesive and around 12–15% for those bonded with 12% adhesive. This substantial swelling reflects the hydrophilic character of lignocellulosic fibers, in which accessible hydroxyl groups readily bond with water, leading to dimensional instability [54,55]. In contrast, Gly-CA modification noticeably reduced the TS. Particleboards modified with 20% Gly-CA showed TS values in the range of 7–10% after 24 h, while 40% Gly-CA modification further decreased the swelling to ~4–6%, irrespective of the immersion time. The improved TS values are primarily attributed to esterification between citric acid (CA) and hydroxyl groups in rattan skin particles and glycerol. This reaction forms crosslinked polyester networks, which reduce hygroscopicity and limit water penetration. Increasing Gly-CA concentration (up to 40%) intensifies the crosslinking, leading to the reduced availability of free -OH groups, decreased capillary water uptake, and improved dimensional stability [50,51]. Additionally, the crosslinked matrix enhances fiber bonding and reduces interparticle voids, further limiting the swelling. The results agree with previous findings on esterified particleboards, where citric acid significantly improved the dimensional stability [8,10,50,51].

The adhesive concentration also influenced the swelling behavior. Panels bonded with 12% adhesive consistently exhibited lower TS than those bonded with 6%. This improvement can be contributed to the denser adhesive network, which better encapsulates rattan skin particles and reduces voids, thus restricting the water penetration. However, in the Gly-CA modified boards, the effect of the adhesive concentration was less pronounced, suggesting that chemical modification played a more dominant role in controlling the thickness stability. Overall, the best performance was observed for particleboards made from 40% Gly-CA modified rattan skin with 12% adhesive, which displayed a TS of only ~4–6% after 24 h immersion. These findings clearly demonstrate the benefits of Gly-CA modification and higher adhesive loading in enhancing dimensional stability under water exposure.

### 3.2. Mechanical Properties

**Figure 6:**
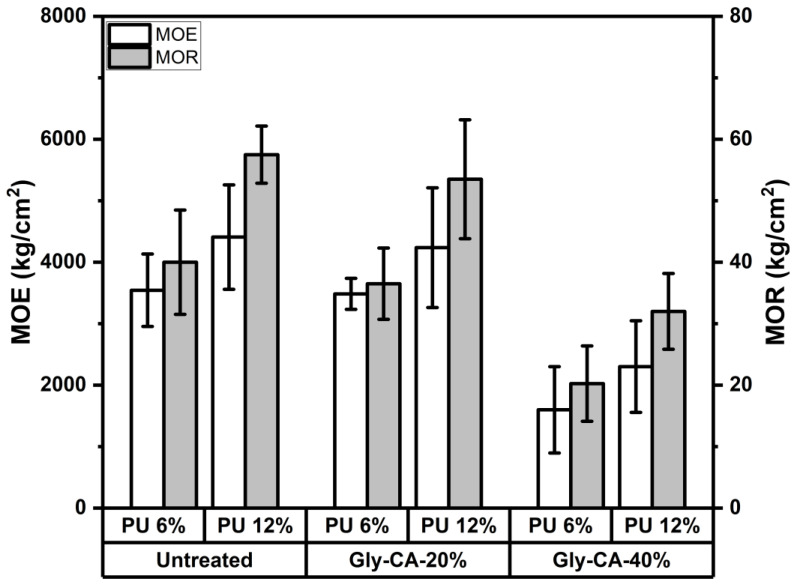
Modulus of elasticity (MOE) and modulus of rupture (MOR) of particleboards made from untreated and Gly-CA-modified rattan skin at different adhesive concentration.

The modulus of elasticity (MOE) and modulus of rupture (MOR) of particleboards produced from untreated and Gly-CA-modified rattan skin are presented in Figure 6. In general, both properties were strongly influenced by the rattan skin modification level and adhesive concentration. Untreated particleboards displayed the highest bending properties, with MOE values of approximately 3500–4500 kg/cm^2^ and MOR values of 45–65 kg/cm^2^, depending on the adhesive loading. These results are consistent with the inherently strong lignocellulosic structure of rattan skin, which provides stiffness and strength in the absence of chemical modification [54]. Gly-CA modification significantly affected the bending performance. Boards modified with 20% Gly-CA retained moderate bending strength, with an MOE in the range of 3000–4500 kg/cm^2^ and an MOR of 35–60 kg/cm^2^, indicating that partial modification can improve the dimensional stability (see the WA and TS results) while maintaining acceptable strength levels. However, the boards prepared with 40% Gly-CA exhibited much lower values, with an MOE reduced to 1500–2500 kg/cm^2^ and an MOR to 20–35 kg/cm^2^. The reduction in bending properties at higher modification levels may be explained by plasticization of the fiber matrix and possible degradation or over-crosslinking of hemicellulose and lignin during citric acid treatment, which limits the effective stress transfer [10,51,55].

The adhesive content also played a crucial role. In all formulations, increasing the adhesive loading from 6% to 12% improved both the MOE and MOR. This improvement can be attributed to the enhanced bonding interface and more efficient stress transfer between particles when sufficient polyurethane adhesive is available. For untreated boards, this increase was most pronounced, while in modified boards, the effect was somewhat less dramatic due to the dominant influence of chemical modification. Overall, the best performance was achieved in untreated boards with 12% adhesive, which provided the highest stiffness and strength. However, this formulation suffers from poor dimensional stability in water. By comparison, 20% Gly-CA modified boards with 12% adhesive offered a better balance, maintaining adequate mechanical performance while significantly improving the water resistance. In contrast, 40% Gly-CA modification, although effective in reducing water absorption and thickness swelling, the compromised bending properties highlight the trade-off between mechanical strength and dimensional stability.

**Figure 7:**
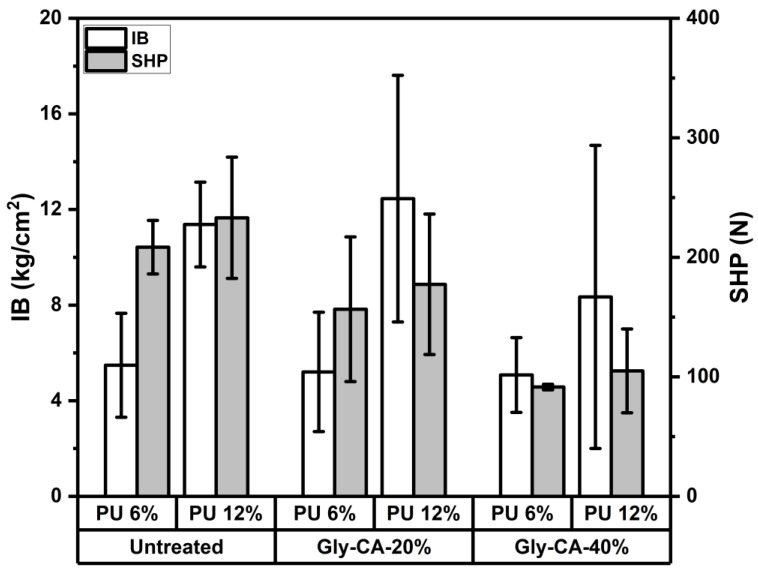
Internal bonding (IB) and screw-holding power (SHP) of particleboards made from untreated and Gly-CA-modified rattan skin at different adhesive concentrations.

The internal bonding (IB) strength of particleboards reflects the adhesive cohesion within the board as well as the interfacial adhesion between the rattan skin particles and the polyurethane resin. As shown in Figure 7, the untreated rattan skin particleboards exhibited the highest IB values, ranging from ~10 to 12 kg/cm^2^, particularly at 12% adhesive loading. This superior performance can be attributed to the abundance of available hydroxyl groups in untreated rattan skin, which enhance polyurethane–wood chemical interactions through hydrogen bonding and urethane linkages [56]. In contrast, Gly-CA modification reduced the IB strength, with values decreasing to ~6–12 kg/cm^2^ for Gly-CA-20% and ~4–8 kg/cm^2^ for Gly-CA-40%. The reduction is most likely due to the esterification of cell wall hydroxyl groups during Gly-CA treatment, which decreases the reactive sites available for bonding with polyurethane. Similar reductions in bonding strength following chemical modification have been reported in esterified and acetylated wood [54,57]. Despite this reduction, Gly-CA-20% boards still maintained moderate IB values, suggesting that partial hydroxyl substitution preserves sufficient reactivity while enhancing dimensional stability. The adhesive concentration significantly influenced the IB strength. Boards bonded with 12% adhesive consistently showed higher IB values than those with 6%, confirming that increased resin loading improves interparticle contact and load transfer as well as internal cohesion in particleboard systems.

The screw-holding power (SHP) results followed a similar trend. Untreated rattan skin boards displayed the highest SHP (~200–250 N), while Gly-CA-20% showed moderate reductions (~150–200 N). The lowest SHP values (~80–130 N) were observed for Gly-CA-40%, particularly at 6% adhesive loading. The decrease in SHP is related to the reduced IB strength and weaker fiber–resin interlocking caused by excessive chemical modification, which alters the cell wall porosity and hinders adhesive penetration [55]. Nevertheless, the boards bonded with 12% adhesive exhibited improved SHP compared to their 6% counterparts, indicating that resin availability remains a dominant factor in enhancing screw anchorage. Overall, these findings indicate a clear trade-off between dimensional stability and mechanical bonding strength. Untreated rattan skin provides superior IB and SHP but poor dimensional stability. Gly-CA-20% modification offers a compromise, retaining adequate IB and SHP while improving the water resistance, whereas Gly-CA-40% significantly sacrifices the bonding performance.

### 3.3. Chemical Properties

#### 3.3.1. Fourier Transform Infrared (FTIR) Analysis

**Figure 8:**
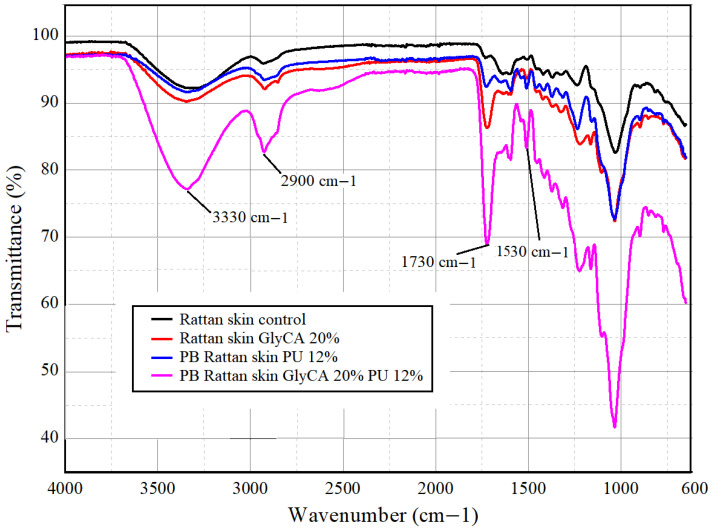
FITR of untreated rattan skin, Gly-CA 20%-impregnated rattan skin, particleboards of untreated rattan skin bonded with PU 12%, and particleboards of Gly-CA 20%-modified rattan skin bonded with PU 12%.

The FTIR spectra of untreated rattan skin, Gly-CA-impregnated rattan skin (20%), particleboard made from untreated rattan skin with 12% PU adhesive, and particleboard made from Gly-CA 20% rattan skin with 12% PU adhesive are presented in Figure 8. The main absorption bands reflect the characteristic functional groups of lignocellulosic components (cellulose, hemicellulose, and lignin) and the introduced ester bonds from glycerol–citric acid modification. The untreated rattan skin spectrum (black line) exhibits typical peaks of lignocellulosic materials: a broad band around ~3330 cm^−1^ corresponding to O–H stretching vibrations of hydroxyl groups, peaks at ~2900 cm^−1^ assigned to C–H stretching of aliphatic groups, and a strong absorption at ~1030–1050 cm^−1^ associated with C–O stretching of cellulose and hemicellulose [58,59]. The band at ~1735 cm^−1^, attributed to unconjugated C=O stretching of hemicellulose acetyl and ester groups, is weak, indicating partial degradation during drying or natural variation. After impregnation (red line), a distinct increase in intensity around 1720–1735 cm^−1^ is observed, confirming the formation of ester linkages between citric acid and the hydroxyl groups of rattan skin cell walls [10,60,61]. Simultaneously, the O–H stretching band at ~3330 cm^−1^ becomes less intense, indicating the consumption of hydroxyl groups during esterification. These changes suggest successful chemical modification by Gly-CA.

The spectrum of particleboard bonded with 12% polyurethane adhesive (blue line) shows new features relative to the untreated rattan skin. A noticeable band near ~1700–1725 cm^−1^ corresponds to urethane C=O stretching vibrations, while the region around ~1530–1550 cm^−1^ indicates N–H bending coupled with C–N stretching [56]. These bands reflect chemical interactions between the isocyanate groups of the adhesive and the hydroxyl groups of rattan skin. The spectrum of the Gly-CA-modified rattan skin particleboard with adhesive (magenta line) exhibits the most pronounced changes. The 1730 cm^−1^ ester band becomes very strong, reflecting overlapping contributions from Gly-CA esterification and urethane linkages. The O–H band around ~3330 cm^−1^ is further reduced, confirming extensive hydroxyl substitution. The C-H stretching of the aliphatic group strongly intensified, confirming the addition of hydrocarbon from the PU adhesive and the Gly-CA. Additionally, more complex features appear in the 1000–1200 cm^−1^ region (C–O and C–O–C stretching), indicating overlapping signals from the ester and urethane bonds. These results demonstrate that both Gly-CA modification and polyurethane adhesive contribute synergistically to chemical crosslinking within the particleboard structure. The FTIR spectra confirm that (i) untreated rattan skin retains abundant hydroxyl groups; (ii) Gly-CA impregnation introduces ester bonds while reducing hydroxyl availability; (iii) polyurethane adhesive introduces urethane linkages; and (iv) combined Gly-CA treatment and adhesive bonding result in intensified ester/urethane bands, reflecting a more chemically crosslinked matrix.

#### 3.3.2. Thermogravimetric Analysis

**Figure 9:**
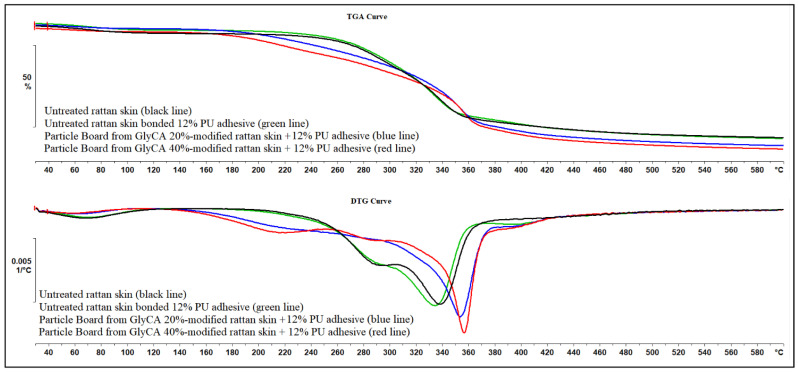
TGA and DTG curves of untreated rattan skin, particleboards of untreated rattan skin bonded with PU 12%, particleboards of Gly-CA 20%-modified rattan skin bonded with PU 12%, and particleboards of Gly-CA 20%-modified rattan skin bonded with PU 12%.

Thermogravimetric analysis (TGA) and differential thermogravimetric (DTG) results of untreated rattan skin, particleboards of untreated rattan skin bonded with PU 12%, particleboards of Gly-CA 20%-modified rattan skin bonded with PU 12%, and particleboards of Gly-CA 20%-modified rattan skin bonded with PU 12% are shown in Figure 9. The thermal degradation behavior of the untreated rattan skin and its particle boards was significantly influenced by both the PU adhesive incorporation and Gly-CA modification (Figure 9; Table 3).

The untreated rattan skin exhibited a primary degradation event with a maximum decomposition temperature (T_max_) at 338.5 °C, corresponding to the thermal scission of cellulose and hemicelluloses, and a total weight loss of ~52.3%. Incorporation of 12% PU adhesive slightly reduced the mass loss (~51.0%) while shifting T_max_ to 334.5 °C, indicating that the adhesive network contributed to increased char formation and restricted volatile release. A more pronounced effect was observed in Gly-CA-modified boards: Gly-CA 20% with PU showed a T_max_ of 353.9 °C with a higher mass loss (~62.9%), while Gly-CA 40% with PU further improved the thermal stability (T_max_ 356.8 °C) but still exhibited elevated mass loss (~59.7%). These findings suggest that Gly-CA modification enhances thermal stability by introducing esterified crosslinking networks within rattan cell walls, which delays the onset of degradation but simultaneously promotes higher volatile release and reduces the char yield. Similar observations of increased T_max_ and altered residue behavior after esterification or crosslinking treatments have been reported for wood and lignocellulosic composites [10]. Overall, PU adhesive mainly enhances the char stability, while Gly-CA modification dominates the thermal profile by raising T_max_ and broadening the degradation range, with the 40% Gly-CA treatment showing the most thermally resistant system.

### 3.4. Field Test Study

**Figure 10:**
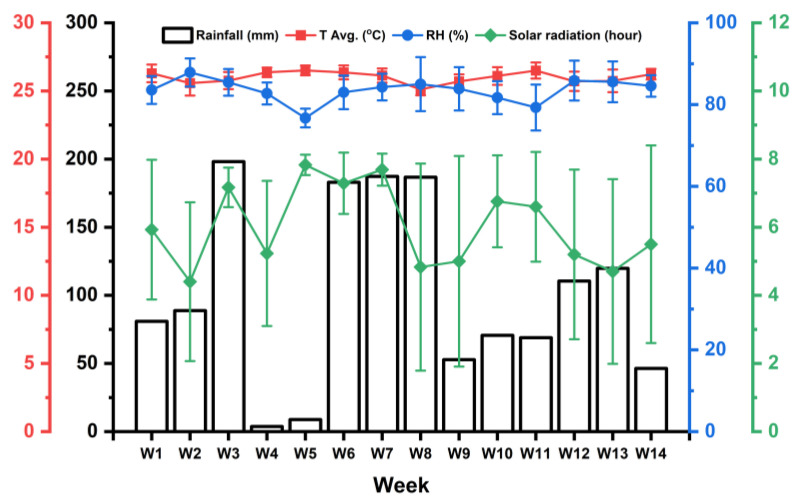
Ombrothermic graph of field test site at Arboretum, Faculty of Forestry and Environment, IPB University, from 21 June to 26 September 2025 (Bureau of Meteorology, 2025) [62].

The termite resistance of particleboards made from untreated and Gly-CA–modified rattan skin at different PU adhesive concentrations was evaluated using a 98-day field exposure (graveyard) test at the Arboretum, Faculty of Forestry and Environment, IPB University, Bogor, Indonesia. Figure 10 shows the weekly climatic conditions during the 14-week field test, including rainfall (mm), average temperature (°C), relative humidity (RH, %), and solar radiation (hours). Throughout the observation period, the average temperature remained relatively stable between 25 and 27 °C, indicating consistent warm tropical conditions. The relative humidity (RH) also remained high, generally fluctuating between 75 and 85%, reflecting a humid environment favorable for fungal and termite activity. The rainfall varied considerably from week to week, with peaks exceeding 200 mm observed during weeks 3, 6, and 7, indicating periods of intense precipitation. Conversely, minimal rainfall was recorded in weeks 4–5 and week 14, showing intermittent dry periods within the overall wet season pattern. The solar radiation showed an inverse trend to rainfall, ranging from 4 to 11 h per day. Higher solar radiation values were observed during weeks with lower rainfall (e.g., weeks 4–5 and 10–11), while intense rainfall periods corresponded to reduced solar exposure (weeks 6–8). In general, the field site experienced warm, humid, and periodically wet conditions throughout the 14-week test. Such an environment provided a realistic and challenging exposure setting for evaluating the durability and termite resistance of the rattan skin-based particleboards.

**Figure 11:**
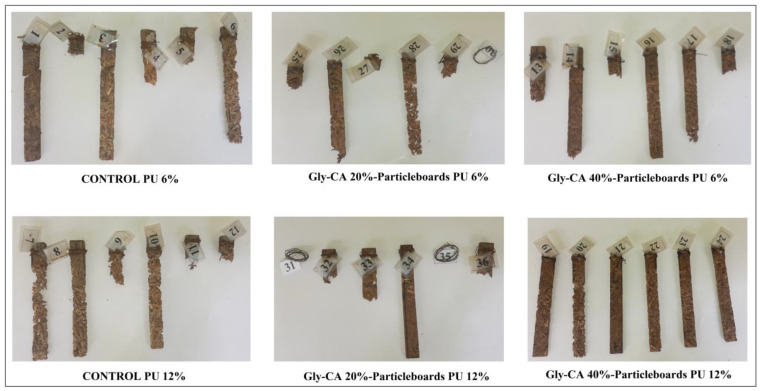
Termite attack appearance of untreated and Gly-CA–modified rattan particleboards after a 3-month field exposure: untreated–PU 6%, Gly-CA 20%–PU 6%, Gly-CA 40%–PU 6%, untreated–PU 12%, Gly-CA 20%–PU 12%, and Gly-CA 40%–PU 12%.

The average mass loss and corresponding termite damage ratings according to the AWPA E7-07 (2008) [53] standard are summarized in Table 4 and illustrated in Figure 11. The results revealed that both chemical modification and adhesive concentration significantly influenced the resistance of the particleboards to termite attack. The untreated particleboards bonded with 6% and 12% PU adhesives exhibited substantial mass losses of 49.3% and 54.7%, respectively, corresponding to a very severe attack (rating 4). This indicates that the polyurethane adhesive alone could not provide adequate protection against termite degradation.

The boards prepared from Gly-CA 20%-modified rattan skin experienced even higher mass losses of 71.6% and 69.4% for the 6% and 12% PU adhesive, respectively, both classified as failure (rating 0). This pronounced susceptibility may be attributed to incomplete esterification or partial depolymerization during mild modification, which might have increased the accessibility of cellulose and hemicellulose to termite enzymatic digestion. In contrast, the Gly-CA 40%-modified rattan skin particleboards showed a substantial reduction in mass loss, particularly for the 12% PU adhesive group, where the average mass loss decreased to 15.3%, corresponding to a moderate attack (rating 8). The 40% modified boards bonded with 6% PU adhesive exhibited 43.9% mass loss (rating 6), indicating a severe but significantly reduced attack compared to the untreated samples. The improved resistance at higher Gly-CA concentrations can be attributed to the increased ester crosslinking between hydroxyl groups of the rattan cell wall polymers and citric acid, forming a more hydrophobic and biologically stable matrix that limits enzymatic degradation and nutrient availability [11,54,55]. From these field test results, we can conclude that the combined effects of higher Gly-CA modification (40%) and stronger adhesive bonding (12%) yielded the highest termite resistance, demonstrating the potential of bio-based esterification and polymer reinforcement in enhancing the biological durability of rattan skin-based composites.

## 4. Conclusions

This study demonstrates that glycerol–citric acid (Gly-CA) modification in combination with polyurethane (PU) adhesive, markedly enhances the dimensional stability, thermal performance, and biological durability of rattan-skin particleboards. Gly-CA treatment effectively reduced the water absorption and thickness swelling, reaching 35–40% and 4–6%, respectively, at 40% Gly-CA with 12% PU, due to esterification that decreases hydrophilicity. The mechanical properties declined at higher modification levels, but 20% Gly-CA provided the best balance between strength retention and water resistance.

Chemical and thermal analyses (FTIR and TGA) confirmed increased crosslinking, formation of ester and urethane bonds, and improved thermal stability (T_max up to 356.8 °C). Importantly, field tests revealed significant gains in termite resistance. While the untreated boards suffered severe attack, the 40% Gly-CA + 12% PU boards showed the lowest mass loss (15.3%) and achieved an AWPA rating of 8, indicating moderate attack only.

In general, Gly-CA modification, especially at 40% with 12% PU adhesive, substantially improves the durability and environmental resistance, while the 20% level provides the most balanced mechanical and physical performance. These findings confirm the potential of Gly-CA bio-modification as a sustainable strategy for developing dimensionally stable and termite-resistant rattan skin-based composites.

## Figures and Tables

**Table 1:** Termite rating scheme according to the AWPA E7-07 (2008) standard.

Rating	Description
10	Sound
9.5	Trace, surface nibbles permitted
9	Slight attack, up to 3% of cross-sectional area affected
8	Moderate attack, 3–10% of cross-sectional area affected
7	Moderate/severe attack and penetration, 10–30% of cross-sectional area affected
6	Severe attack, 30–50% of cross-sectional area affected
4	Very severe attack, 50–75% of cross-sectional area affected
0	Failure

**Table 2:** Color characteristics of particleboard.

Treatment	Resin Content	L*	a*	b*	∆E
Untreated	6%	65.20 (2.19)	6.60 (2.19)	33.40 (3.21)	-
	12%	64.20 (1.64)	1.64 (8.20)	8.20 (1.64)	-
Gly-CA-20%	6%	41.80 (1.30)	1.30 (17.2)	17.20 (1.30)	26.55 (6.06)
	12%	36.80 (3.78)	3.78 (17.4)	17.40 (3.78)	29.29 (3.75)
Gly-CA-40%	6%	23.00 (1.48)	1.48 (14.2)	14.20 (1.48)	46.02 (6.12)
	12%	23.60 (2.07)	2.07 (14.6)	14.60 (2.07)	42.66 (5.43)

Remarks: L* for lightness (0 = black, 100 = white), a* for the green-red spectrum (−128 to 127, green to red), b* for the blue-yellow spectrum (−128 to 127, blue to yellow); Values in parentheses are standard deviations.

**Table 3:** TGA and DTG data of untreated rattan skin, particleboards of untreated rattan skin bonded with PU 12%, particleboards of Gly-CA 20%-modified rattan skin bonded with PU 12%, and particleboards of Gly-CA 20%-modified rattan skin bonded with PU 12%.

Sample	TGA: Mass Loss (%)	TGA: Decomposition Range (°C)	TGA: T_max_ (°C)	DTG: Peak Temp (°C)	Notes
Untreated Rattan Skin (Black line)	−52.30%	130.2–443.0	338.5	326.1	Typical lignocellulosic profile; hemicellulose + cellulose decomposition
PB (Untreated Rattan Skin + PU 12%) (Green line)	−51.00%	106.9–420.3	334.5	325.1	PU adhesive increases char, slightly improves stability
PB (Gly-CA 20% + PU 12%) (Blue line)	−62.90%	103.5–487.7	353.9	349.9	Crosslinking raises T_max_ but reduces char residue
PB (Gly-CA 40% + PU 12%) (Red line)	−59.70%	72.8–559.6	356.8	351.8	Highest T_max_, most stable system; secondary peak from modified domains

**Table 4:** Mass loss values due to field test and termite damage rating of untreated and Gly-CA-modified rattan particleboards after a 3-month field exposure.

Treatment	Mass Loss (%)	Termite Rating (AWPA E7-07)	Description
Untreated–PU 6%	49.3 ± 28.7	4	Very severe attack (50–75% of cross-sectional area affected).
Untreated–PU 12%	54.7 ± 25	4	Very severe attack (50–75% area affected).
Gly-CA 20%–PU 6%	71.6 ± 29.2	0	Failure—nearly complete deterioration.
Gly-CA 20%–PU 12%	69.4 ± 35.4	0	Failure—very high degradation.
Gly-CA 40%–PU 6%	43.9 ± 27.9	6	Severe attack (30–50% affected).
Gly-CA 40%–PU 12%	15.3 ± 9.8	8	Moderate attack (3–10% affected).

## Data Availability

The original contributions presented in this study are included in the article. Further inquiries can be directed to the corresponding authors.
